# Supplementary material for: Upregulated pexophagy limits the capacity of selective autophagy
Source: Nat Commun. 2024 Jan 9;15:375. doi: 10.1038/s41467-023-44005-4 (PMC10776696; doi:10.1038/s41467-023-44005-4)
Supplement: Supplementary file 1 — Supplementary Information [file 41467_2023_44005_MOESM1_ESM.pdf]

Supplementary Information

Upregulated pexophagy limits the capacity of selective autophagy.

Authors and Affiliations:

Kyla Germain,[1,2] Raphaella W. L. So, [2,3] Laura F. DiGiovanni,[1,2] Joel C. Watts,[2,3] Robert H. J. Bandsma,[4,5,*] Peter K. Kim[1,2,*]

[1] Cell Biology Program, The Hospital for Sick Children, Toronto, ON M5G 1X8, Canada

[2] Department of Biochemistry, University of Toronto, Toronto, ON M5S 1A8, Canada

[3] Tanz Centre for Research in Neurodegenerative Diseases, Toronto, ON M5T 0S8, Canada

[4] Translational Medicine Program, The Hospital for Sick Children, Toronto, ON M5G 1X8, Canada

[5] Department of Nutritional Sciences, Faculty of Medicine, University of Toronto, ON M5S 1A8, Canada

* Co-Correspondence: pkim@sickkids.ca; robert.bandsma@sickkids.ca

Supplementary Table 1: List of Reagents Used.

Reagent	Commercial Supplier
**KITS**	
SV Total RNA Isolation Program	Promega
High-Capacity cDNA Reverse Transcription	Applied Biosystems
TaqMan Fast Advanced Master Mix	Applied Biosystems
Pierce^{TM} BCA Protein Assay Kit	Thermo Fisher Scientific
**CHEMICALS**	
Oligomycin	Fisher Scientific; AAJ61898MA
Antimycin A1	Santa Cruz; sc-202467
Puromycin	BioShop; PUR333
Rapamycin	BioShop; RAP004
3-Hydroxy-1,2-dimethyl-4(1H)-pyridone (DFP)	Sigma-Aldrich; 379409
Carbonyl cyanide 3-chlorophenylhydrazone (CCCP)	Sigma-Aldrich; C2759
MRT68921 dihydrochloride (LYN-1604)	Sigma-Aldrich; SML1644
Bafilomycin A1	Santa Cruz; sc-201550A
Torin1	Abcam; ab218606
Leupeptin	BioShop; LEU001
E-64	Millipore Sigma; E3132
Cresyl Violet Acetate	Sigma-Aldrich; C5042
Concanamycin A	Abcam; ab144227
Dimethyl sulfoxide (DMSO)	Sigma-Aldrich; D8418

Supplementary Table 2: List of siRNAs Used.

Gene Target	RNA sequence (5' – 3')
PEX1	CCAAGCAACUUCAGUCAAA
PEX2	CUCUUACUGGUGCACCUAA
PEX13	CGGUAUCUUUACAGACGGCUAC
PEX14	GAACUCAAGUCCGAAAUU
ATG12	GUGGGCAGUAGAGCGAACA
NBR1	GGAGUGGAUUUACCAGUUAUU
FIP200	AACACUGGAACUACUCUAACA
HTT	AACUUUCAGCUACCAAGAAAG
Non-targeting control	AAUAAGGCUAUGAAGAGAUAC

Supplementary Table 3: List of Antibodies Used.

Target	Product Number	Dilution	
		Immuno-fluorescence	Western Blot
**PRIMARY ANTIBODIES**			
PMP70	Abcam; ab3421	1:2000	1:1000
PEX1	BD Biosciences; 611719		1:250
PEX13	Abcam; ab96841		1:1000
PEX14	Proteintech; 10594-1-AP		1:5000
ATG12	Cell Signaling; 2010		1:500
NBR1	Abnova; H00004077-MOI		1:500
MAP1LC3B	Thermo Fisher Scientific; A-21070	1:1000	1:1000
SQSTM1	BD Biosciences; 610832		1:1000
OPTN	Novus Biologicals; NBPP1-84682		1:1000
CVα	Abcam; ab14748		1:5000
MFN2	Abcam; ab56889		1:1000
HSP60	Abcam; ab46798	1:2000	1:10,000
FK2	Enzo; BML-PW8810	1:200	
FK1	Millipore Sigma; 04-262	1:200	
LAMP1A	DSHB; H5C6	1:1000	
ATG13	Cell Signaling; 13468		1:1000
phos-ATG13	Thermo Fisher Scientific; 600-401-C49S		1:1000
BNIP3L/Nix	Cell Signaling; 12396		1:1000
mTOR	Cell Signaling; 2983		1:1000
phos-mTOR	Cell Signaling; 2971		1:1000
p70 S6K	Cell Signaling; 2708		1:1000
phos-p70 S6K	Cell Signaling; 9205		1:1000
FIP200	Cell Signaling; 12436		1:1000
FLAG (WB)	Millipore Sigma; F3165		1:8000
FLAG (IF)	Millipore Sigma; F7425	1:1000	
α-Synuclein	BD Biosciences; 610786	1:1000	
Catalase	Millipore Sigma; 219010		1:5000
HTT	Millipore Sigma; MAB2166	1:1000	1:1000
ULK1	Cell Signaling; 8054		1:1000
phos-ULK1	Cell Signaling; 14202		1:1000
**SECONDARY ANTIBODIES**			
GAPDH-HRP	Novus Biologicals; NB300-328H		1:10,000
β-Actin-HRP	Cell Signaling; 5125		1:10,000
Vinculin-HRP	Cell Signaling; 18799		1:10,000
anti-rabbit IgG-HRP	Thermo Scientific; 31460		1:10,000
anti-mouse IgG-HRP	Cedarlane; CLCC30007		1:10,000
**a**nti-mouse IgG Alexa Fluor 488	Thermo Fisher Scientific; A11001	1:1000	

anti-rabbit IgG Alexa Fluor 568	Thermo Fisher Scientific; A-11011	1:1000
anti-mouse IgG Alexa Fluor 568	Thermo Fisher Scientific; A-11004	1:1000
anti-mouse IgG Alexa Fluor 647	Thermo Fisher Scientific; A-31571	1:1000

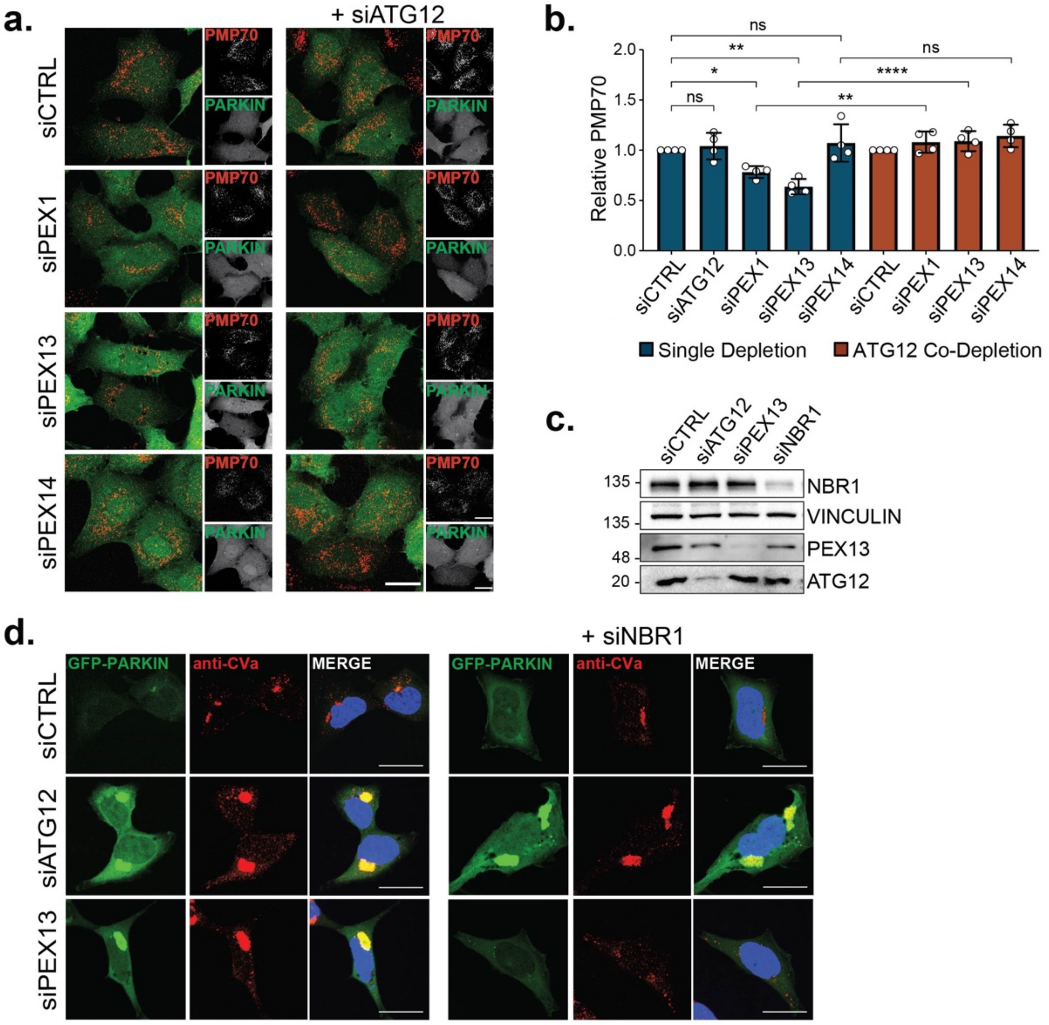

Supplementary Data Figure 1. PEX1 or PEX13 depletion increases pexophagy and impairs mitophagy in GFP-Parkin HEK cells. (a) Immunofluorescent images of GFP-Parkin HEK cells treated with the indicated siRNA and immunostained for peroxisomal marker, PMP70. Scale bar, 25μm. **(b)** Quantification of PMP70 in (a) relative to siCTRL conditions. PMP70 was measured by dividing the number of PMP70 puncta by cell volume. Data are displayed as means ± standard deviation from *n*=4 independent experiments. Statistical significance is denoted as * *P* ≤0.05, ** *P* ≤ 0.01, One-way ANOVA, Tukey's multiple comparisons test. **(c)** Immunoblot of GFP-Parkin HEK cells treated with the indicated siRNA and probed for NBR1, PEX13, and ATG12. **(d)** Representative images of GFP-Parkin HEK cells treated with the indicated siRNA(s) prior to treatment for 8-h with 2.5μM Oligomycin and 250nM Antimycin A1. Cells were immunostained for mitochondria marker, CVα. Scale bars, 25μm. Source data and exact *P* values are provided as a Source Data file.

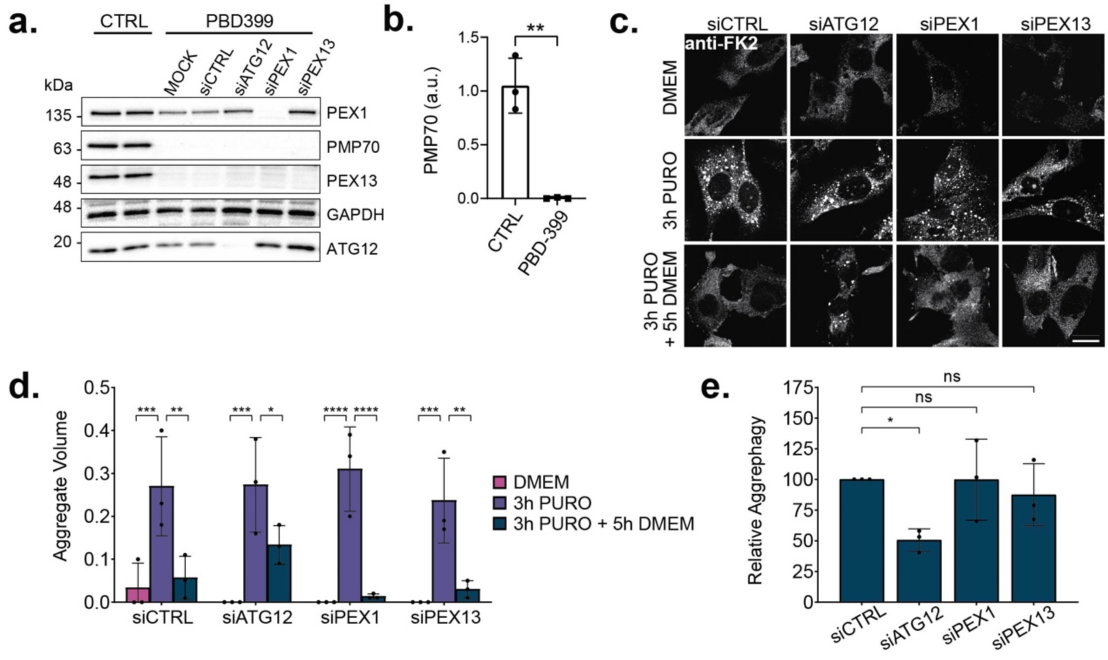

Supplementary Data Figure 2. Peroxisome-deficient fibroblasts have unimpaired aggrephagy. (a) Immunoblot of Control and PBD399-T1 fibroblast cells treated with siRNA and probed for the indicated proteins. **(b)** Densitometry quantification of PMP70 bands normalized to loading control GAPDH. **(c)** PBD399-T1 fibroblasts treated with the indicated siRNA prior to aggrephagy induction. Representative images from cells at each stage in the assay: DMEM, 3-h 5μg mL^{-1} Puromycin, or 3-h 5μg mL^{-1} Puromycin followed by 5-h clearance period in DMEM. Cells were immunostained for FK2. Scale bars, 25μm. **(d)** Quantification of Relative Aggregate Volume in (c). Relative Aggregate Volume was calculated by dividing the total volume of FK2 puncta by cell volume. **(e)** Quantification of aggrephagy activity in (c), relative to siRNA-control-transfected cells. Aggrephagy activity was measured as the % clearance of FK2 aggregates. Data are displayed as means ± standard deviation from n=3 independent experiments. Statistical significance is denoted as *$P \leq 0.05$, **$P \leq 0.01$, ***$P \leq 0.001$, ****$P \leq 0.0001$, ns not significant. (b) Unpaired t-test, two tailed. (d) Two-way ANOVA, Tukey's multiple comparisons test. (e) One-way ANOVA, Dunnett's multiple comparisons test. Source data and exact P values are provided as a Source Data file.

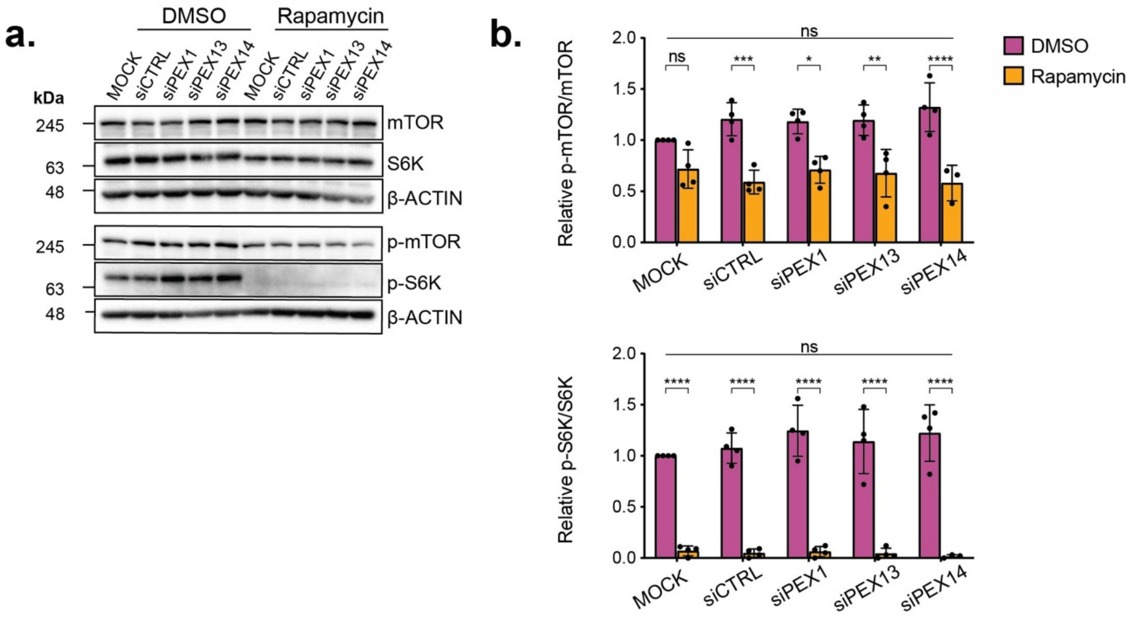

Supplementary Data Figure 3. Rapamycin treatment inhibits mTOR in PEX1 and PEX13 depleted cells. (a) Immunoblots of HeLa cells treated with the indicated siRNA and grown in either DMSO or 2μM Rapamycin for 5-h. Immunoblots were probed for mTOR, SK6, phospho-mTOR, phospho-SK6, and loading control β-Actin. Quantification of **(b)** mTOR and **(c)** S6K phosphorylation from (a), relative to MOCK. mTOR and S6K phosphorylation were calculated by dividing the band intensity of phospho-mTOR/mTOR or phospho-SK6/S6K. Band intensities were first measured using ImageJ software and normalized to their respective β-Actin loading control. Data are displayed as means ± standard deviation from n=4 independent trials. Statistical significance is denoted as *$P \leq 0.05$, **$P \leq 0.01$, ***$P \leq 0.001$, ****$P \leq 0.0001$, ns not significant, Two-way ANOVA, Tukey's multiple comparisons test. Source data and exact P values are provided as a Source Data file.

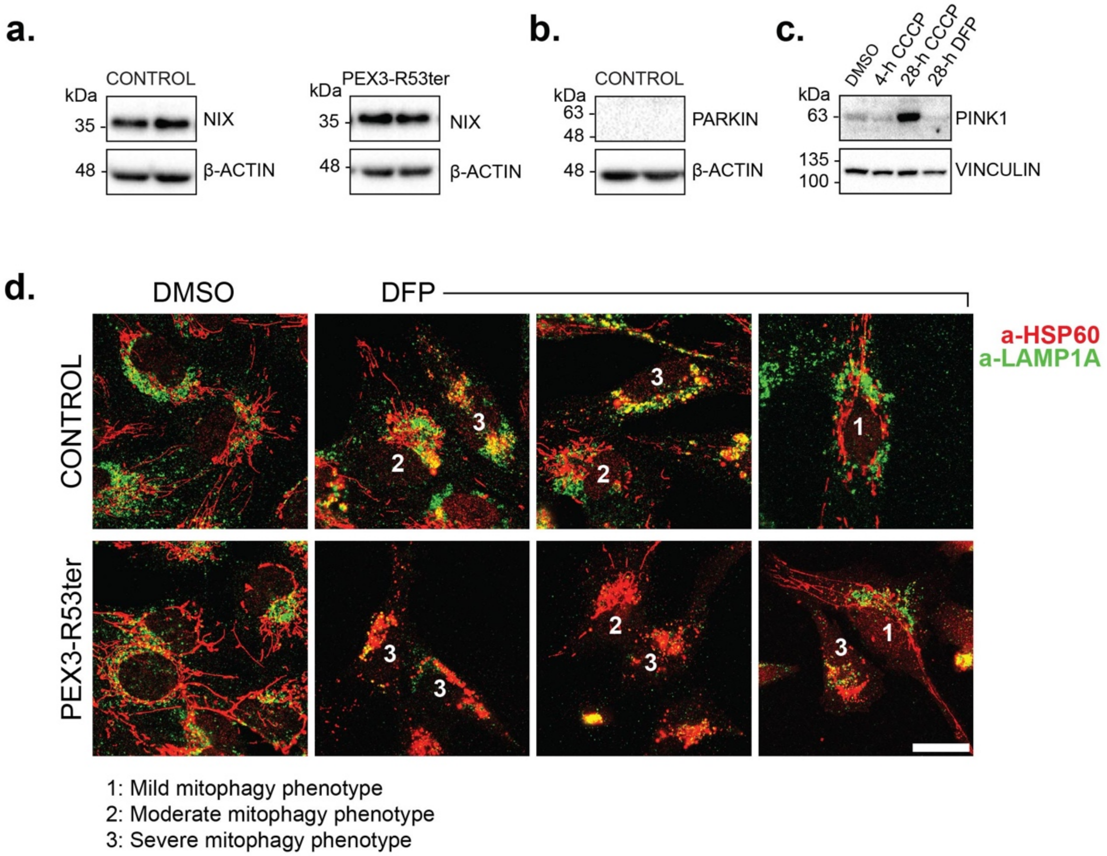

Supplementary Data Figure 4. Deferiprone induces mitophagy phenotype in human fibroblasts. (a) Immunoblots of control or PEX3-R53Ter ZSD fibroblasts probed for NIX and loading control, β-Actin. **(b)** Immunoblots of control fibroblasts probed for Parkin and β-Actin. **(c)** Immunoblots of control fibroblasts treated with either CCCP or DFP for the indicated time and probed for PINK1 and VINCULIN. **(d)** Representative images of control or PEX3-R53Ter ZSD fibroblasts treated for 28-h with either DMSO or 1mM DFP and labelled with mitophagy phenotype (corresponding to quantification in Fig. 4j). Cells were immunostained for HSP60 and LAMP1A. Scale bars, 25μm.

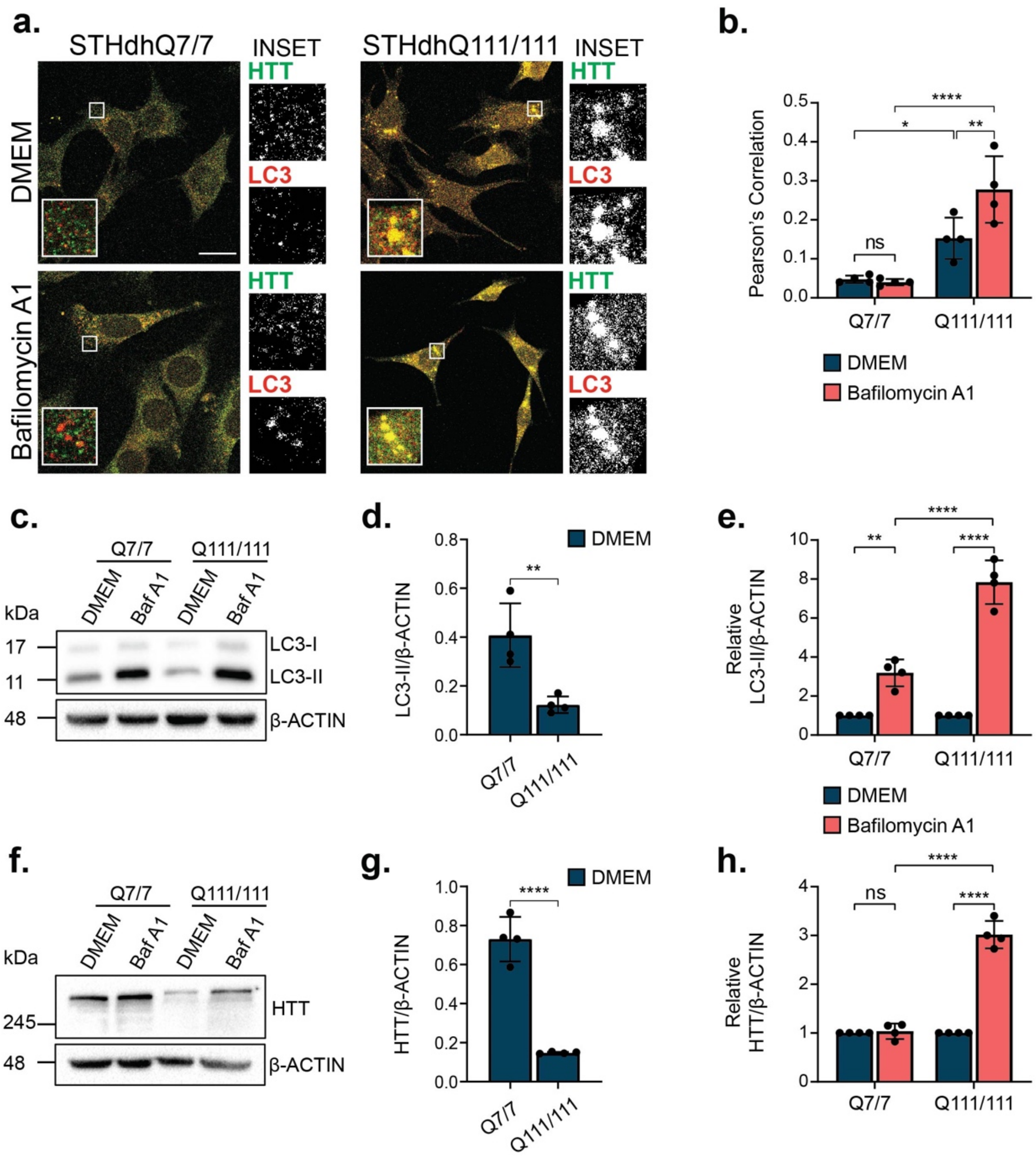

a.
STHdhQ7/7
INSET
STHdhQ111/111
INSET
HTT
LC3
DMEM
Bafilomycin A1
b.
Pearson's Correlation
0.5
0.4
0.3
0.2
0.1
0.0
****
*
**
ns
Q7/7
Q111/111
DMEM
Bafilomycin A1
c.
Q7/7
Q111/111
DMEM
Baf A1
kDa
17
11
48
LC3-I
LC3-II
β-ACTIN
d.
DMEM
LC3-II/β-ACTIN
0.8
0.6
0.4
0.2
0.0
**
Q7/7
Q111/111
e.
Relative LC3-II/β-ACTIN
10
8
6
4
2
0
**
****
****
Q7/7
Q111/111
DMEM
Bafilomycin A1
f.
Q7/7
Q111/111
DMEM
Baf A1
kDa
245
48
HTT
β-ACTIN
g.
DMEM
HTT/β-ACTIN
1.0
0.8
0.6
0.4
0.2
0.0
****
Q7/7
Q111/111
h.
Relative HTT/β-ACTIN
4
3
2
1
0
ns
****
****
Q7/7
Q111/111

Supplementary Data Figure 5. STHdh$^{Q111/111}$ cells undergo increased HTT turnover by autophagy. (a) Immunofluorescent images of STHdh$^{Q7/7}$ or STHdh$^{Q111/111}$ cells grown in DMEM or 0.25mM Bafilomycin A1 for 24-h and immunostained for HTT and LC3. Scale bar, 25μm. White squares denote zoomed region. **(b)** Pearson's correlation coefficient for LC3 and HTT from (a). **(c)** Immunoblot of STHdh$^{Q7/7}$ or STHdh$^{Q111/111}$ cells grown in DMEM or 0.25mM Bafilomycin A1 for 24-h and probed for **(c)** LC3 or **(f)** HTT. Quantification of **(d)** LC3-II or **(g)** HTT bands normalized to β-Actin for DMEM condition. Quantification of normalized **(e)** LC3-II or **(h)** HTT bands relative to DMEM condition for each cell type. Data are displayed as means from n=4 independent experiments. Statistical significance is denoted as $*P \leq 0.05$, $**P \leq 0.01$, $***P \leq 0.001$, $****P \leq 0.0001$, ns not significant. (b, e, h) Two-way ANOVA, Šidák's multiple comparison text. (d, g) Unpaired t-test, two tailed. Source data and exact P values are provided as a Source Data file.

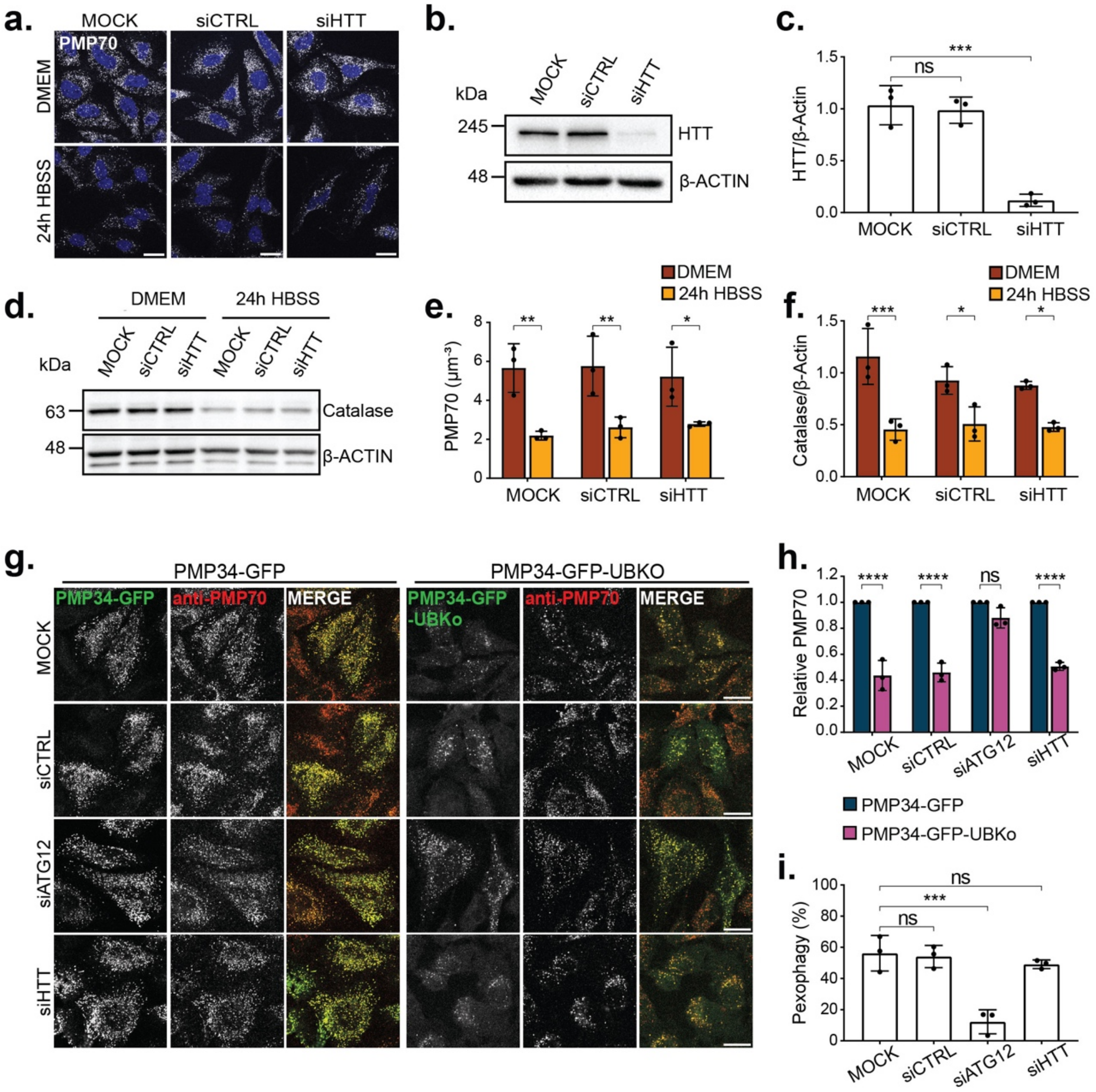
a.
MOCK
siCTRL
siHTT
PMP70
DMEM
24h HBSS
b.
kDa
MOCK
siCTRL
siHTT
245
48
HTT
β-ACTIN
c.
HTT/β-Actin
ns
***
MOCK
siCTRL
siHTT
d.
DMEM
24h HBSS
kDa
63
48
Catalase
β-ACTIN
e.
PMP70 (μm⁻³)
DMEM
24h HBSS
f.
Catalase/β-Actin
g.
PMP34-GFP
PMP34-GFP-UBKO
PMP34-GFP
anti-PMP70
MERGE
PMP34-GFP -UBKo
siATG12
h.
Relative PMP70
PMP34-GFP
PMP34-GFP-UBKo
i.
Pexophagy (%)

Supplementary Data Figure 6. HTT depletion does not influence pexophagy. (a) Immunofluorescent images of HeLa cells treated with the indicated siRNA and grown in either DMEM or HBSS for 24-h. Cells are immunostained for PMP70, blue=DAPI; Scale bars, 25μm. **(b)** Immunoblot of HeLa cells treated with the indicated siRNA and probed for HTT and β-Actin. **(c)** Quantification of HTT bands normalized to β-Actin. **(d)** Immunoblot of HeLa cells treated with the indicated siRNA and grown in either DMEM or HBSS for 24-h and probed for Catalase. **(e)** Quantification of PMP70 in (a). PMP70 was measured by dividing the number of PMP70 puncta by cell volume. **(f)** Quantification of Catalase bands normalized to β-Actin. **(g)** Immunofluorescent images of HeLa cells treated with the indicated siRNA and transiently expressing either PMP34-GFP or PMP34-GFP-UBKo for 48-h. Cells are immunostained for PMP70, blue=DAPI; Scale bars, 25μm. **(h)** Quantification of PMP70 in **(g)**, relative to PMP34-GFP expressing cells for each siRNA condition. **(i)** Quantification of pexophagy in (g). Pexophagy was quantified as percentage loss of PMP70 with PMP34-GFP-UBKo expression compared to PMP34-GFP expression. Data are displayed as means from *n*=3 independent experiments $\pm$ standard deviation. Statistical significance is denoted as *$P \leq 0.05$, **$P \leq 0.01$, ***$P \leq 0.001$, ****$P \leq 0.0001$, ns not significant. (c, i) One-way ANOVA, Dunnett's multiple comparison test. (e, f, g) Two-way ANOVA, Tukey's multiple comparisons test. Source data and exact *P* values are provided as a Source Data file.

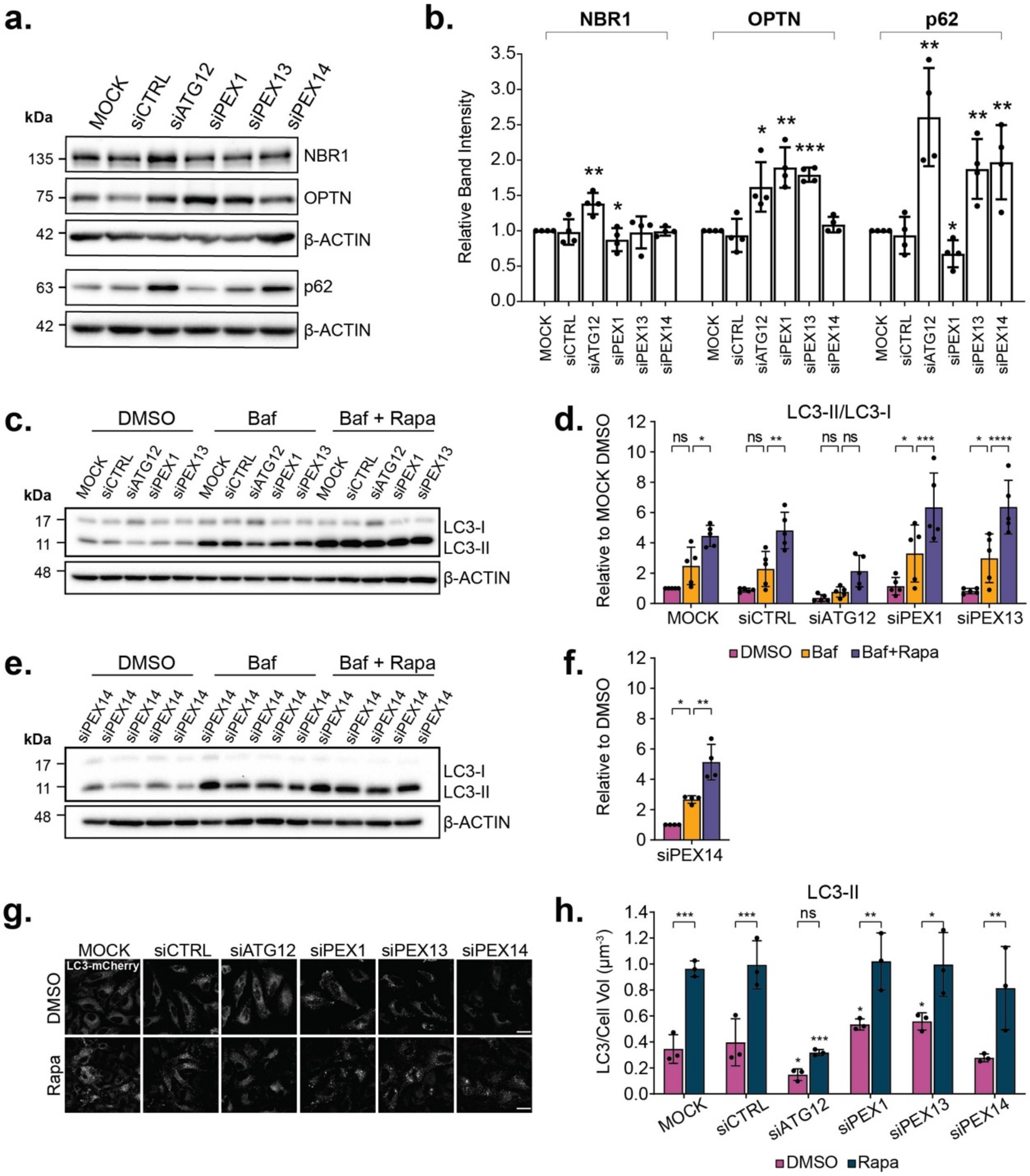
a.
b.
NBR1
OPTN
p62
Relative Band Intensity
MOCK
siCTRL
siATG12
siPEX1
siPEX13
siPEX14
kDa
β-ACTIN
c.
d.
LC3-II/LC3-I
DMSO
Baf
Baf + Rapa
LC3-I
LC3-II
Relative to MOCK DMSO
e.
f.
Relative to DMSO
g.
h.
LC3-II
LC3-mCherry
Rapa
LC3/Cell Vol (μm⁻³)
Baf+Rapa

Supplementary Data Figure 7. Rapamycin treatment induces autophagy flux in PEX1 and PEX13 depleted cells. (a) Immunoblots of HeLa cells treated with the indicated siRNA and probed for NBR1, OPTN, p62 and loading control β-Actin. **(b)** Densitometry quantification of NBR1, OPTN, and p62 bands in (a) normalized to loading control and relative to MOCK. **(c)** Immunoblot of HeLa cells treated with the indicted siRNA and grown in either DMSO, 200nM Bafilomycin, or 2μM Rapamycin + 200nM Bafilomycin A1 for 5-h. **(d)** Densitometry quantification of LC3-II/LC3-I ratio in (c), relative to MOCK DMSO condition. **(e)** Immunoblot of HeLa cells treated with siRNA targeting PEX14 and grown in either DMSO, 200nM Bafilomycin, or 2μM Rapamycin + 200nM Bafilomycin A1 for 5-h. 4 lanes per condition in the immunoblot correspond to samples from *n*=4 independent trials. **(f)** The mean densitometry quantification of LC3-II/LC3-I ratio in (e), relative to DMSO condition. **(g)** Representative images of HeLa cells treated with the indicated siRNA, transfected with LC3-mCherry, and grown in either DMSO or 2μM Rapamycin for 5-h. Cells were digitonin permeabilized prior to fixation. Scale bars, 25μm. **(h)** Quantification of the volume of LC3 puncta, relative to cell volume in (g). Data represent means from *n*=4 (b, f), *n*=5 (d), or *n*=3 (h) independent experiments ± standard deviation. Statistical significance is denoted as *$P \leq 0.05$, **$P \leq 0.01$, ***$P \leq 0.001$, ****$P \leq 0.0001$, ns not significant. (b, h) Unpaired *t*-test, two tailed to MOCK. (d, h) Two-way ANOVA, Tukey's multiple comparisons test. (f) One-way ANOVA, Tukey's multiple comparisons test. Source data and exact *P* values are provided as a Source Data file.

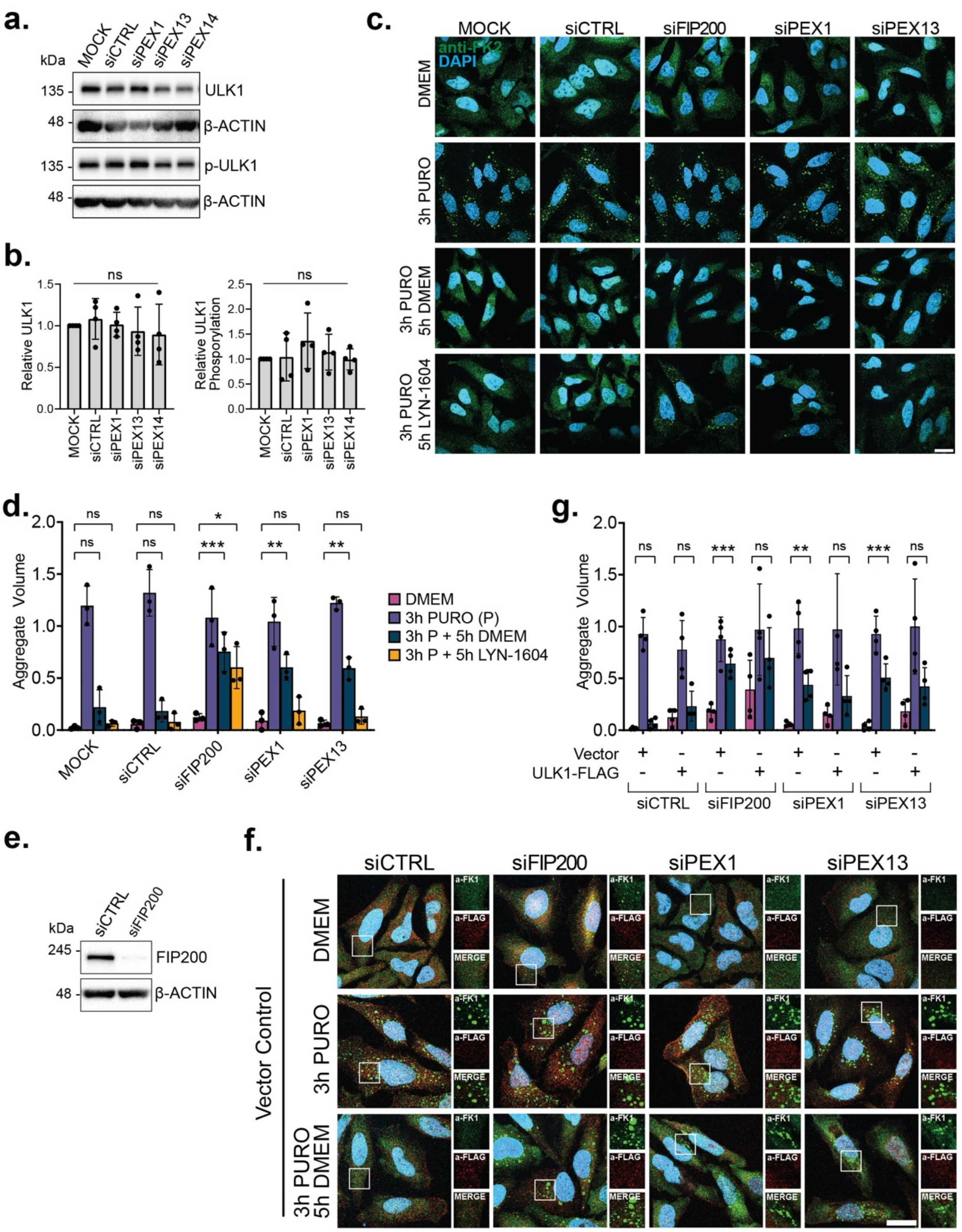

a.
kDa
MOCK
siCTRL
siPEX1
siPEX13
siPEX14
135
ULK1
48
β-ACTIN
135
p-ULK1
48
β-ACTIN
b.
ns
Relative ULK1
Relative ULK1 Phosporylation
MOCK
siCTRL
siPEX1
siPEX13
siPEX14
c.
MOCK
siCTRL
siFIP200
siPEX1
siPEX13
anti-FK2
DAPI
DMEM
3h PURO
3h PURO 5h DMEM
3h PURO 5h LYN-1604
d.
Aggregate Volume
ns
*
***
**
DMEM
3h PURO (P)
3h P + 5h DMEM
3h P + 5h LYN-1604
MOCK
siCTRL
siFIP200
siPEX1
siPEX13
g.
Aggregate Volume
Vector
ULK1-FLAG
siCTRL
siFIP200
siPEX1
siPEX13
e.
kDa
siCTRL
siFIP200
245
FIP200
48
β-ACTIN
f.
siCTRL
siFIP200
siPEX1
siPEX13
Vector Control
DMEM
3h PURO
3h PURO 5h DMEM
a-FK1
a-FLAG
MERGE

Supplementary Data Figure 8. Impaired aggrephagy during PEX1 or PEX13 depletion can be rescued by ULK1 activation: extended data. (a) Immunoblots of HeLa cells treated with the indicated siRNA and probed for ULK1, p-ULK1, and loading control β-Actin. **(b)** Quantification of total ULK1 and ULK1 phosphorylation from (a), relative to MOCK. ULK1 phosphorylation was calculated by dividing the band intensity of phospho-ULK1/ULK1. Band intensities were first measured using ImageJ software and normalized to their respective β-Actin loading control. **(c)** Representative images from cells at each stage in the assay: DMEM, 3-h 5μg mL^{-1} Puromycin, 3-h 5μg mL^{-1} Puromycin followed by 5-h clearance period in DMEM, or 3-h 5μg mL^{-1} Puromycin followed by 5-h clearance period in 20μM LYN-1604. Cells were immunostained with the ubiquitin antibody FK2; blue=DAPI. Scale bars, 25μm. **(d)** Quantification of Aggregate Volume in (c). Aggregate Volume was calculated by dividing the total volume of FK2 puncta by cell volume. **(e)** Immunoblot of HeLa cells treated with control non-targeting or FIP200-targeting siRNA and probed for the indicated proteins. **(f)** Representative images of HeLa cells treated with the indicated siRNA prior to transfection of an empty vector and subjection to the puromycin-aggrephagy assay. Cells were immunostained for FK2 and FLAG; blue=DAPI. Scale bars, 25μm; white boxes, zoomed region. **(g)** Quantification of Aggregate Volume in (e, Fig. 8f). Data are displayed as means from *n*=3 (d) or *n*=4 (a, g) independent experiments ± standard deviation. (b) One-way ANOVA, Dunnett's multiple comparisons test. (d, g) Two-way ANOVA, Tukey's multiple comparisons test. Source data and exact *P* values are provided as a Source Data file.
