# Supplementary material for: Upregulated pexophagy limits the capacity of selective autophagy
Source: Nat Commun. 2024 Jan 9;15:375. doi: 10.1038/s41467-023-44005-4 (PMC10776696; doi:10.1038/s41467-023-44005-4)
Supplement: Supplementary file 3 — Reporting Summary [file 41467_2023_44005_MOESM3_ESM.pdf]

nature portfolio

Corresponding author(s): Peter K. Kim and Robert Bandsma

Last updated by author(s): Nov 19, 2023

Reporting Summary

Nature Portfolio wishes to improve the reproducibility of the work that we publish. This form provides structure for consistency and transparency in reporting. For further information on Nature Portfolio policies, see our Editorial Policies and the Editorial Policy Checklist.

Statistics

For all statistical analyses, confirm that the following items are present in the figure legend, table legend, main text, or Methods section.

n/a	Confirmed	
☐	☒	The exact sample size (*n*) for each experimental group/condition, given as a discrete number and unit of measurement
☐	☒	A statement on whether measurements were taken from distinct samples or whether the same sample was measured repeatedly
☐	☒	The statistical test(s) used AND whether they are one- or two-sided<br>*Only common tests should be described solely by name; describe more complex techniques in the Methods section.*
☒	☐	A description of all covariates tested
☐	☒	A description of any assumptions or corrections, such as tests of normality and adjustment for multiple comparisons
☐	☒	A full description of the statistical parameters including central tendency (e.g. means) or other basic estimates (e.g. regression coefficient) AND variation (e.g. standard deviation) or associated estimates of uncertainty (e.g. confidence intervals)
☐	☒	For null hypothesis testing, the test statistic (e.g. *F*, *t*, *r*) with confidence intervals, effect sizes, degrees of freedom and *P* value noted<br>*Give P values as exact values whenever suitable.*
☒	☐	For Bayesian analysis, information on the choice of priors and Markov chain Monte Carlo settings
☒	☐	For hierarchical and complex designs, identification of the appropriate level for tests and full reporting of outcomes
☐	☒	Estimates of effect sizes (e.g. Cohen's *d*, Pearson's *r*), indicating how they were calculated

Our web collection on statistics for biologists contains articles on many of the points above.

Software and code

Policy information about availability of computer code

Data collection	Zeiss Zen Blue (Zeiss), ChemiDoc Imaging System (Bio-Rad Laboratories).
Data analysis	Volocity (Version 6.3.1), FIJI, Prism-GraphPad (Version 10.1.0), Zeiss Zen Blue

For manuscripts utilizing custom algorithms or software that are central to the research but not yet described in published literature, software must be made available to editors and reviewers. We strongly encourage code deposition in a community repository (e.g. GitHub). See the Nature Portfolio guidelines for submitting code & software for further information.

Data

Policy information about availability of data

All manuscripts must include a data availability statement. This statement should provide the following information, where applicable:

- Accession codes, unique identifiers, or web links for publicly available datasets
- A description of any restrictions on data availability
- For clinical datasets or third party data, please ensure that the statement adheres to our policy

Source data for Figures 1-9 and Supplementary Data Figures 1-8 are provided as a Source Data file. A reporting summary is also available in the Supplementary information. All microscopy image datasets that support the findings of this manuscript are available from the corresponding authors upon reasonable request.

Research involving human participants, their data, or biological material

Policy information about studies with human participants or human data. See also policy information about sex, gender (identity/presentation), and sexual orientation and race, ethnicity and racism.

Reporting on sex and gender	N/A
Reporting on race, ethnicity, or other socially relevant groupings	N/A
Population characteristics	N/A
Recruitment	N/A
Ethics oversight	N/A

Note that full information on the approval of the study protocol must also be provided in the manuscript.

Field-specific reporting

Please select the one below that is the best fit for your research. If you are not sure, read the appropriate sections before making your selection.

[x] Life sciences [ ] Behavioural & social sciences [ ] Ecological, evolutionary & environmental sciences

For a reference copy of the document with all sections, see nature.com/documents/nr-reporting-summary-flat.pdf

Life sciences study design

All studies must disclose on these points even when the disclosure is negative.

Sample size	No sample size calculation was done. Instead, at least three independent experiments (n =3) were conducted for all experiments, a standard practice for experiments using cultured cells. The number of independent experiments is stated in the legends of all figures. The mean calculation for each independent experiment was calculated from 25-40 cells. Similarly, the representative images are from 3 to 4 independent experiments where 25-40 cells were imaged per experiment.
Data exclusions	No data were excluded from the analyses.
Replication	At least 3 independent trials were performed of each experiment to ensure reproducibility.
Randomization	The experiments were not randomized in this study, as the same culture cells were used for a given experiment. All cells started from the same initial source before being deliberately treated with different conditions (siRNA or drug treatment). This study has no drug efficacy or safety studies in animals or humans.
Blinding	The experimenter was not blinded. Blinding was not necessary because the analysis were analyst independent. The only exception was mitophagy phenotype scoring in Fig 4j where both the microscopist and individual quantifying were blinded.

Reporting for specific materials, systems and methods

We require information from authors about some types of materials, experimental systems and methods used in many studies. Here, indicate whether each material, system or method listed is relevant to your study. If you are not sure if a list item applies to your research, read the appropriate section before selecting a response.

Materials & experimental systems

n/a	Involved in the study	
[ ]	[x]	Antibodies
[ ]	[x]	Eukaryotic cell lines
[x]	[ ]	Palaeontology and archaeology
[x]	[ ]	Animals and other organisms
[x]	[ ]	Clinical data
[x]	[ ]	Dual use research of concern
[x]	[ ]	Plants

Methods

n/a	Involved in the study	
[x]	[ ]	ChIP-seq
[x]	[ ]	Flow cytometry
[x]	[ ]	MRI-based neuroimaging

Antibodies

Antibodies used	See Supplementary Information Table 3 for this information.
Validation	See Supplementary Information Table 3 for this information.

Eukaryotic cell lines

Policy information about cell lines and Sex and Gender in Research

Cell line source(s)	HeLa cells were purchased from American Type Culture Collection (CCL-2).<br>Stably transfected GFP-Parkin HEK293 cells were a gift from Dr. Angus McQuibban (University of Toronto, Toronto, ON, Canada). The immortalized patient-derived skin fibroblast cell lines from healthy (Control), PEX1-G843D, and PEX3-R53ter human fibroblast cells were gifts from Dr. Nancy Braverman (McGill University, Montreal, QC, Canada) and PEX19- deficient (PBD399-T1) immortalized human fibroblast was a gift from S.J Gould (John Hopkins University School of Medicine, Baltimore, MD). STHdhQ111/111 and STHdhQ7/7 cells were donated by Dr. Ray Truant (McMaster University, Hamilton, ON, Canada).
Authentication	PBD cell lines were previously confirmed by sequencing (reference indicated). Also we have validated the peroxisome phenotype associated with these PBD cell lines by image analysis of immunofluorescence staining . The STHdhQ111/111 cells were confirmed to express mutant HTT by western blot, which is shown in the Results section of the manuscript. No further genomic sequencing were done.
Mycoplasma contamination	All cells used were mycoplasma negative. Cells were routinely tested for mycoplasma using the PCR based detection assay from FroggaBio, product number: 25235. All cells were discarded after 25-30 passages and new cell tested for mycoplasma were used.
Commonly misidentified lines<br>(See ICLAC register)	None.
